# Bioinspired Tannic Acid-Modified Coffee Grounds as Sustainable Fillers: Effect on the Properties of Polybutylene Adipate Terephthalate Composites

**DOI:** 10.3390/polym15132769

**Published:** 2023-06-21

**Authors:** Jiaxin Wang, Dong Zhao, Guodong Jiang, Yong Wu, Yucai Shen, Tingwei Wang

**Affiliations:** 1 College of Materials Science and Engineering, Nanjing Tech University, Nanjing 211816, China; m18357330035@163.com (J.W.); dongzhao@njtech.edu.cn (D.Z.); gdjiang@njtech.edu.cn (G.J.); wangtw@njtech.edu.cn (T.W.); 2 Nanjing Wurui Biodegradable New Material Research Institute Co., Ltd., Nanjing 211816, China; 18752096596@163.com

**Keywords:** coffee grounds, tannic acid, PBAT, interfacial adhesion

## Abstract

Preparing composites from gricultural waste with biodegradable polymers is one of the strategies used to ensure the long-term sustainability of such materials. However, due to the differences in their chemical properties, biomass fillers often exhibit poor interfacial adhesion with polymer matrices. Inspired by mussel foot silk, this work focused on the surface modification of coffee grounds (CGs) using a combination of tannic acid (TA) and alkali treatment. CGs were used as a biomass filler to prepare polybutylene adipate terephthalate (PBAT)/CG composites. The modification of CGs was demonstrated by Fourier transform infrared spectroscopy (FTIR), the water contact angle, and scanning electron microscopy (SEM). The effect of CGs on the rheological, tensile, and thermal properties of the PBAT/CG composites was investigated. The results showed that the addition of CGs increased the complex viscosity, and the surface modification enhanced the matrix–filler adhesion. Compared with unmodified CG composites, the tensile strength and the elongation at break of the composite with TA-modified alkali-treated CGs increased by 47.0% and 53.6%, respectively. Although the addition of CGs slightly decreased the thermal stability of PBAT composites, this did not affect the melting processing of PBAT, which often occurs under 200 °C. This approach could provide a novel method for effectively using biomass waste, such as coffee grounds, as fillers for the preparation of polymer composites.

## 1. Introduction

As a popular drink, coffee has gradually become an indispensable part of people’s lives. Based on the latest statistics of the International Coffee Organization (ICO), the global consumption of coffee is approximately 10 million tons [1]. Thus, coffee grounds (CGs) are generated in huge quantities after coffee processing, accounting for more than 50% of coffee beans [2,3]. Currently, they are mostly disposed of by being stored and incinerated; this produces methane and carbon dioxide, which are very harmful to the environment [4,5]. Therefore, there is an urgent need to develop new strategies to address the environmental pollution and disposal difficulties associated with CGs. In this context, many researchers have focused on valorizing CGs to obtain high-value products, such as biofuels [6,7,8], adsorbents [9,10], antioxidants [11,12,13], etc. Furthermore, as a biomass filler, they not only reduce the cost of producing biodegradable polyesters, but also preserve the degradable properties of the composite [14,15]. Research on eco-friendly biomass-based composites has recently become a hot topic.

However, their different chemical properties constitute a real barrier to the incorporation of CGs in polymer matrices, thereby leading to the deterioration of the composites’ properties. Previous studies that have considered the modification of CGs focus on oil extraction [16], alkali treatment [17,18,19], heat treatment [20], silane coupling agents [21], or the addition of third components such as plasticizers [22] and compatibilizers [23]. While ester groups in coffee oil may also cause CGs to agglomerate, the mechanisms of oil extraction, alkali treatment, and heat treatment are mainly reflected in the removal of lipids, hemicellulose, and other small molecules from CGs to improve their dispersibility and specific surface area. Wu et al. [24] found that the mechanical properties of CG/polypropylene (PP) composites were slightly improved after oil extraction. Tang et al. [25] reported that the tensile strength of CG composites treated with 10 wt% sodium hydroxide (NaOH) increased by 12.61% compared to polyhydroxyalkanoates (PHA)/CG composites. Moustafa et al. [26] utilized torrefied CGs as reinforcing agents for polybutylene adipate terephthalate (PBAT). However, these methods have certain limitations, and the CG content usually does not exceed 20 wt%. Plasticizers and compatibilizers are also beneficial for improving the interfacial bonding between the CGs and the polymer matrix. The addition of polyethylene glycol (PEG) as a plasticizer could enhance the compatibility and mechanical properties of PBAT/CG composites [22]. Fang et al. [27] successfully synthesized a polymethyl methacrylate-polyglycidyl methacrylate random (PMMA-r-PGMA) copolymer as a compatibilizer, which effectively enhanced the interfacial interaction between the CGs and poly (butylene succinate) (PBS), but there was an upper limit for the additives and, once the value was exceeded, the material became brittle. Some toxic solvents were also used in the preparation of the compatibilizers. Therefore, a green and efficient CG modification method is needed to obtain high-performance, fully biodegradable CG-filled composites.

Marine mussels can tightly attach to the surfaces of reefs, ships, and other materials under the impact of huge waves, showing remarkable adhesive properties. Biologists have discovered that the secret of mussels’ ability to adhere to substrates is the action of 3,4-dihydroxyphenylalanine [28,29]. The phenolic hydroxyl groups of the polyphenol structure are highly reactive, thus providing strong interfacial interactions. Inspired by mussels, we contend that using substances containing polyphenolic hydroxyl groups could be a simple and easy method for the functionalized modification of the material surface. Tannic acid (TA) is a plant polyphenol that has a strong coordination with metal ions and can adhere to various substrates. Accordingly, the abundance of phenolic groups in TA has the potential to improve adhesion with other materials via hydrogen bonds or hydrophobic interactions [30,31,32]. For example, functionalizing carbon nanotubes (CNTs) with TA decreased CNTs’ agglomeration and enhanced their distribution in a polyamide matrix [33]. Moreover, an ultra-high-molecular-weight polyethylene (UHMWPE) fiber modified by TA improved the surface wettability, roughness, and activity, thereby enhancing the interface adhesion between the fibers and the epoxy resin [34].

In this study, CG was synergistically modified by NaOH and TA. The complexes formed by TA and metal ions (Na^+^) were deposited on the CGs’ surface to increase their roughness and enhance their interfacial adhesion with PBAT. The modified CGs were thoroughly characterized to verify the surface modification strategy. In addition, the fracture morphology, rheological, thermal, and tensile properties, and thermal stability of neat PBAT and PBAT composites were systematically investigated. This work provides a novel, green, and sustainable modification strategy to achieve the efficient utilization of CGs as a biomass filler.

## 2. Materials and Methods

### 2.1. Materials

PBAT (TH801T) was acquired from Blue Ridge Tunhe Chemical Industry Co., Ltd. (Changji, China). Coffee grounds were bought from Shenzhen Xianhe Investment Development Co., Ltd. (Shenzhen, China). Tris (hydroxymethyl) aminomethane (Tris) and tannic acid (TA) were provided by Shanghai Meryer Chemical Technology Co., Ltd. (Shanghai, China). Sodium chloride (NaCl) and sodium hydroxide (NaOH) were obtained from Shanghai Macklin Biochemical Co., Ltd. (Shanghai, China).

### 2.2. Methods

#### 2.2.1. Preparation of CG

The CG was dried in oven at 80 °C and then pulverized into powder by mechanical crusher (800Y, Yongkang Boou Hardware Products Co., Ltd., Yongkang, China). The CG powders were sieved with 100 meshes and collected.

#### 2.2.2. Surface Modification of CG

**Figure 1:**
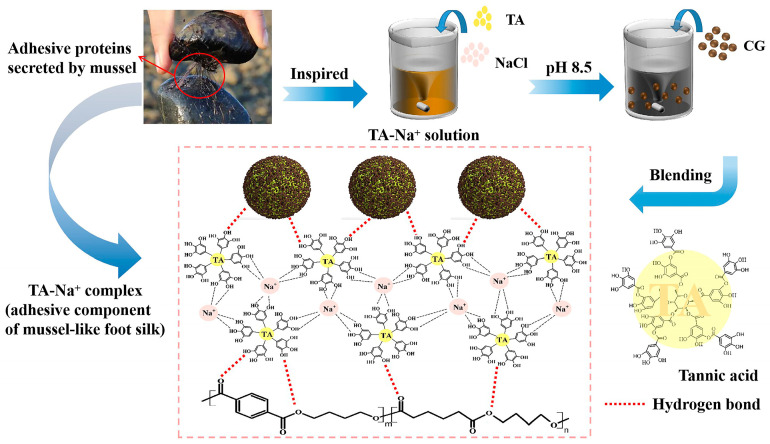
Mechanism diagram of tannic acid-(TA)-surface-modified coffee ground (CG).

The 4.0 g TA and 30.0 g NaCl were dissolved in 2 L of distilled water to achieve 2 g/L TA solution, and the pH was adjusted to 8.5 through Tris. After that, 100.0 g CG was added to TA solution by stirring at room temperature for 24 h. The modified CG was acquired by extraction, washing, drying, and recorded as CG-TA. The mechanism diagram of TA-surface-modified CG is shown in Figure 1. TA forms tris-complex with metal ions at pH > 7. In this paper, the TA-Na^+^ complexes were deposited on the surface of CG, introducing highly reactive polyphenol hydroxyl groups through hydrogen bonding [28,32]. The adhesion component of mussel foot silk protein was simulated on the CG surface, which was expected to enhance the interfacial interaction with the polymer matrix. CG-OH was obtained by impregnating dried CG in 2 wt% NaOH solution for 24 h, and CG-OH-TA was modified by TA on the basis of CG-OH.

#### 2.2.3. Preparation of PBAT/CG Composites

CG and PBAT were fully dried, weighed by 30:70 (*w*/*w*), and manually mixed well. The mixture was melt blended through internal mixer at temperatures of 140 °C for 7 min and the speed of the screws was 60 rpm. The PBAT/CG blends were compression molded at 140 °C with a holding pressure of 10 MPa for 5 min. All composites were fast cooled between two platens.

### 2.3. Characterization

The absorption peaks of CG-related functional groups were recorded by Fourier Transform Infrared Spectroscopy (FTIR) (Nicolet 6700, American Thermo Company) under transmission mode using the potassium bromide compression method. The spectra were gained by 32 scans with resolution of 4 cm^−1^ in the wavenumber range of 4000 to 500 cm^−1^.

The water contact angle was tested at room temperature with contact angle tester (DSA100, Germany). The CG powders were pressed into sheets by infrared sheet press with water as the liquid phase.

The scanning electron microscopy (SEM) (JSM-6510, Japan) was used to observe the surface morphology of CG powder and the frozen fracture morphology of PBAT/CG composites at 10 kV accelerating voltage. Prior to observing the phase morphology, the samples were gold sprayed to enhance conductivity. The elemental content of CG surface could be obtained using Energy-dispersive X-ray spectroscopy (EDS) (OXFORD INCA250, Oxford, UK).

The rheological properties of the neat PBAT and its composites were investigated through rotary rheometer (MCR302, Graz, Austria). Fixed strain values of 1% were used to verify the linear viscoelastic zone. The dynamic frequency scanning was performed at 160 °C and 0.1–100 rad/s shear frequency with fixed strain amplitude. All samples were circular plates that were 25 mm in diameter and 1.0 mm in thickness.

The thermal properties of PBAT and composites were determined using differential scanning calorimetry (DSC) (DSC 204fl Phoenix). All samples (5–8 mg) were heated from 25 °C to 200 °C at a rate of 10 °C/min, held constant for 2 min, and cooled to 25 °C at the same rate. The whole process was under N_2_ atmosphere. The crystallinity (*X_c_*) of PBAT was obtained according to Equation (1).

(1) $$X_{c}\;(\%)=\frac{\Delta H_{m}}{\Delta H_{m}^{0}\times \omega_{PBAT}}\times 100\%$$

where Δ*H_m_^0^* and Δ*H_m_* were the melt enthalpy of 100% crystalline PBAT (114 J·g^−1^) and the melt enthalpy of composites, respectively. *ω_PBAT_* was the mass fraction of PBAT in the composites.

According to ASTM D882-2018, the tensile properties of composite sheets were tested with universal testing machine (CMT5254, Shenzhen Sans Measurement Technology Co., Ltd., Shenzhen, China). For each formulation, the five dumbbell-type specimens were tested with stretching rate of 50 mm/min.

Thermogravimetric analysis (TGA) (Q500, TA Instruments, New Castle, DE, USA) was carried out to determine the thermal stability of PBAT and composites. The experiments were performed in the temperature range of 25–600 °C under N_2_ atmosphere at heating rate of 10 °C/min.

## 3. Results and Discussion

### 3.1. Characterization of CG

**Figure 2:**
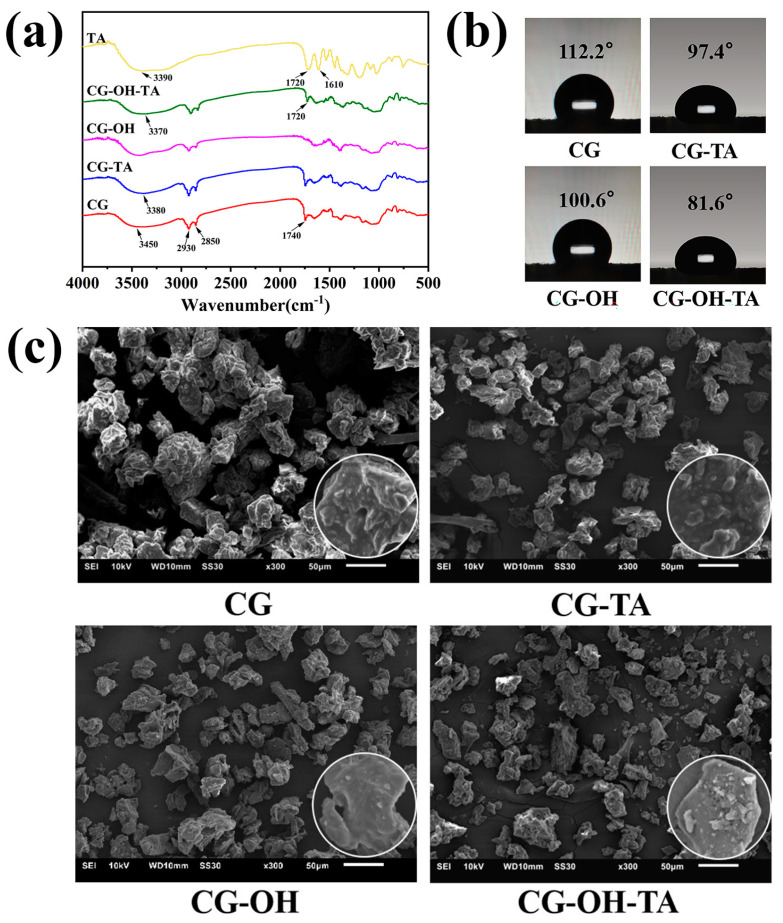
(**a**) FTIR spectra; (**b**) water contact angle; (**c**) SEM images of CG and modified CG.

In order to demonstrate that TA effectively modified CGs and interacted with the CG surface, FTIR was performed to gain insight into the functional groups of TA, pristine CG, and modified CG (Figure 2a). The results show that TA has strong absorption peaks at 3390 cm^−1^ and 1720 cm^−1^, which are attributed to the stretching vibrations of hydroxyl (–OH) and carbonyl groups (C=O), respectively [35]. As for CGs, the appearance of a broad peak in the wavelength range of 3000 to 3600 cm^−1^ corresponds to the stretching vibrations of the O–H and N–H groups present in the lignocellulosic components and proteins. The two sharp peaks near 2850 cm^−1^ and 2930 cm^−1^ correspond to the symmetric and asymmetric stretching of C–H bonds, respectively. Combined with the carbonyl peak at 1740 cm^−1^, these two peaks are associated with ester groups in lipids [36,37]. Since the characteristic peak of TA overlapped with CGs, it was necessary to perform an alkali pretreatment of the CGs. The results showed that the carbonyl group peak of CG-OH disappeared, while the carbonyl group peak of CG-OH-TA reappeared at 1720 cm^−1^ and was consistent with the position of the TA carbonyl peak.

According to Moraczewski [38], the shifting or broadening of hydroxyl groups’ peak position was usually a sign of hydrogen bond formation. Moreover, Guan et al. [39] believed that the stretching vibrations of the hydroxyl group appearing at a lower wavenumber reflected the presence of hydrogen bonding interactions, which was observed in TA-modified CGs. Indeed, the peak position shifted from 3450 cm^−1^ for CG to 3380 cm^−1^ for CG-TA and 3370 cm^−1^ for CG-OH-TA, revealing the formation of new hydrogen bonds between CG and TA.

In addition to FTIR, the results of the contact angle, SEM, and the EDS analysis of CGs and modified CGs are displayed in Figure 2b,c and Table 1. Due to the high lipid content in CGs and interparticle interactions [36], it resulted in CG particle agglomeration and a water contact angle value of 112.2°. The alkali treatment mainly removed lipids and disrupted the adhesion between CG particles; therefore, the structure of CG-OH became loose, and the water contact angle value decreased to 100.6°. The TA modification of CGs did not only improve the compact particle structure, but it also significantly reduced the contact angle value. This phenomenon is due to the modification process and the molecular structure of TA. Indeed, stirring facilitated the dispersion of CG particles, and TA introduced sufficient polar groups on the CG surface to give activity as well as to form TA-Na^+^ complexes, which increased the roughness of the CG surface [34], thus improving the interfacial adhesion. Furthermore, the hydrophilic-modified CGs could improve the interfacial adhesion with the hydrophobic PBAT matrix. In addition, a higher O/C ratio implies a higher TA content on the CG surface [40,41]. The O/C ratio of pristine CGs was 42.2% and increased to 47.5% for CG-TA and 53.9% for CG-OH-TA. The increase in Na, Cl content, and O/C ratio proves the successful modification of CGs by TA.

### 3.2. Characterization of PBAT/CG Composites

#### 3.2.1. Morphology

**Figure 3:**
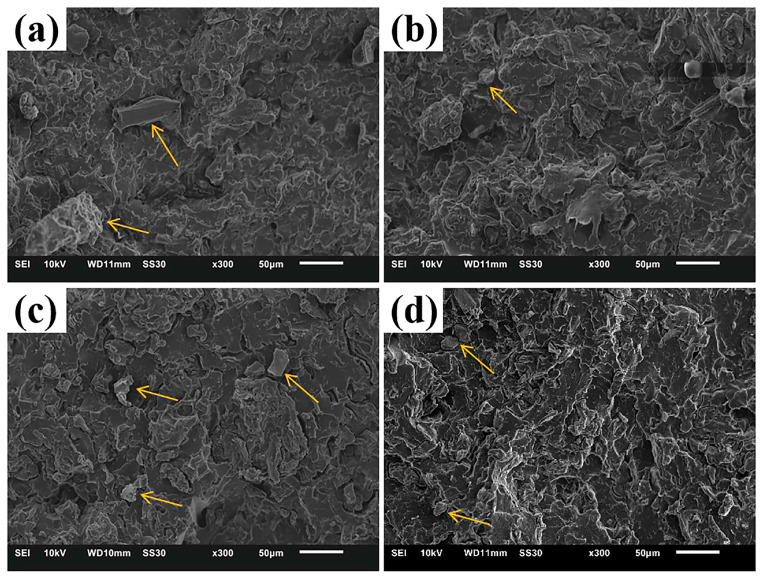
SEM images of (**a**) PBAT/CG; (**b**) PBAT/CG-TA; (**c**) PBAT/CG-OH; and (**d**) PBAT/CG-OH-TA.

By observing the fracture morphologies of PBAT/CG composites acquired by quenched liquid nitrogen, SEM revealed the dispersion and interfacial adhesion of CG in the PBAT matrix (Figure 3). As seen in the fracture morphology of PBAT/CG composites, the addition of CG increased the heterogeneity of PBAT blends owing to the different sizes of CG particles and uneven dispersion [42]. The formation of larger CG particles was attributed to CG self-agglomeration.

In general, the existence of CG decreased the tensile properties of composites in various ways. There were several explanations for this phenomenon. Firstly, the incorporation of CGs interrupted the continuity of PBAT chains and decreased the amount of the matrix, which obstructs the stress transfer and decreases the stress support [43]. Secondly, the CG agglomeration causes stress concentrations, creating weak points in the composites [42]. Moreover, Obasi [44] suggested that the decrease in mechanical properties of composites was also related to the polarity difference between the filler and polymer matrix and their poor interfacial adhesion.

SEM results show that the fracture morphologies of PBAT/CG-TA and PBAT/CG-OH composites exhibited smaller-sized CG particles than PBAT/CG composites; however, exposed CG particles were still observed. Furthermore, CG-OH-TA significantly enhanced the dispersion and mechanical adhesion of CG in the PBAT matrix. Additionally, no evidence of pull-out or separation of CG particles was observed in fracture morphology of the PBAT/CG-OH-TA composite.

#### 3.2.2. Rheological Properties

**Figure 4:**
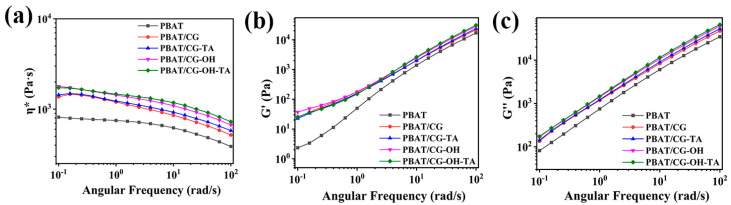
Rheological properties of PBAT and PBAT/CG composites:(**a**) η*; (**b**) G′; and (**c**) G″.

The rheological properties of PBAT and its composites were analyzed to confirm the adhesion of CGs in the PBAT matrix. All samples were in a completely molten state at 160 °C. The complex viscosity (η*) of PBAT appeared to plateau at low and medium frequencies, while it exhibited shear thinning with increasing frequency. This phenomenon was more significant for the composite samples. Furthermore, the incorporation of CGs would hinder the movement of PBAT molecular chains [45]. Compared to the PBAT/CG composite, the η* of modified-CG composites slightly increased, which shows that the modification enhanced the adhesion of CGs and PBAT. As shown in Figure 4, CGs have a reinforcing effect in the PBAT matrix, as the η*, storage modulus (G′), and loss modulus (G″) of PBAT/CG composites were higher than those of neat PBAT [46]. Under the action of TA, the interfacial adhesion between CGs and PBAT is due to the hydrogen bonds and the interfacial adhesion with weak interaction forces. Therefore, the η*, G′, and G″ of modified composites did not significantly increase in rheological curves.

#### 3.2.3. Thermal Properties

**Figure 5:**
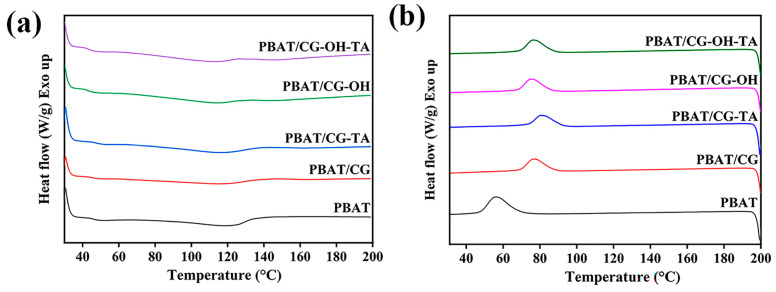
DSC curves from (**a**) the first heating and (**b**) the cooling of PBAT and PBAT/CG composites.

Figure 5 shows the DSC results of PBAT and PBAT/CG composites, and Table 2 summarizes the specific thermal property data. The heating curve of neat PBAT displayed an onset melting temperature of 97.4 °C and a low crystallinity of 8.3%. The incorporation of CGs did not induce any obvious effect on the melting temperature (T_m_) of PBAT, while the crystallization temperature (T_c_) shifted from 55.6 °C for neat PBAT to higher temperatures (74–76 °C). This shift suggests that CGs may be acting as nucleating agents [47]. The crystallinity of PBAT/CG composites was slightly lower compared to neat PBAT, which may be explained by the enhanced adhesion between modified CGs and PBAT, which hinders the mobility of PBAT chains.

#### 3.2.4. Tensile Properties

**Figure 6:**
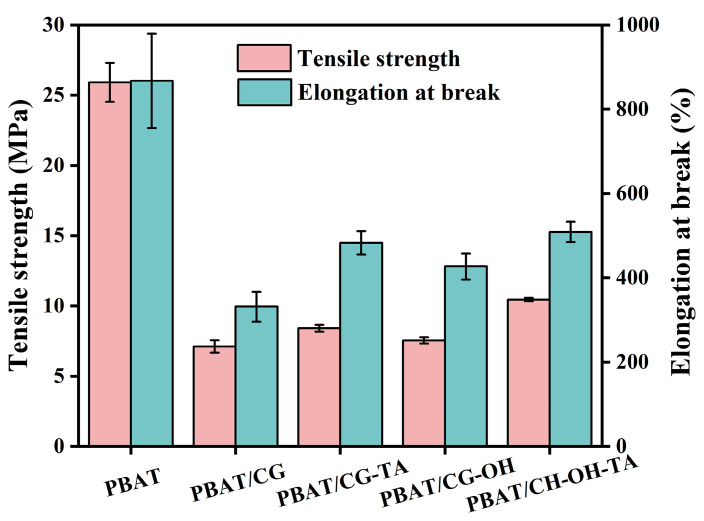
The tensile properties of PBAT and PBAT/CG composites.

The tensile properties of neat PBAT and PBAT composites are shown in Figure 6. PBAT has excellent tensile properties with good tensile strength (~25.9 MPa) and an excellent elongation at break (~867.1%). In contrast, the direct incorporation of 30 wt% CGs into PBAT would reduce the overall tensile properties of the prepared composites. Indeed, the tensile strength and elongation at the break of the PBAT/CG composite decreased to 7.1 MPa and 331.2%, respectively. This may be explained by the agglomeration of CGs, the weak interfacial adhesion of CG in PBAT matrix, and reductions in the continuous region of PBAT, as shown in SEM images.

Although the PBAT/modified CG composite exhibited worse tensile properties compared to neat PBAT, these properties were still considerably improved compared to PBAT/CG composite. Firstly, the surface treatment weakened the intermolecular interaction of CGs. Secondly, alkali treatment disrupted the interparticle adhesion of CGs and promoted their migration [48]. In comparison with the PBAT/CG composite, the tensile strength and elongation at break of PBAT/CG-OH-TA composite increased by 47.0% and 53.6%, respectively. The alkali-treated CG surface favors the TA-Na^+^ complexes deposition and increases the CGs’ surface roughness. Moreover, TA-Na^+^ complexes can act as the interface, interlocking pins to generate higher friction with CG and PBAT, which also contributed to the enhancement of interfacial adhesion [34].

**Figure 7:**
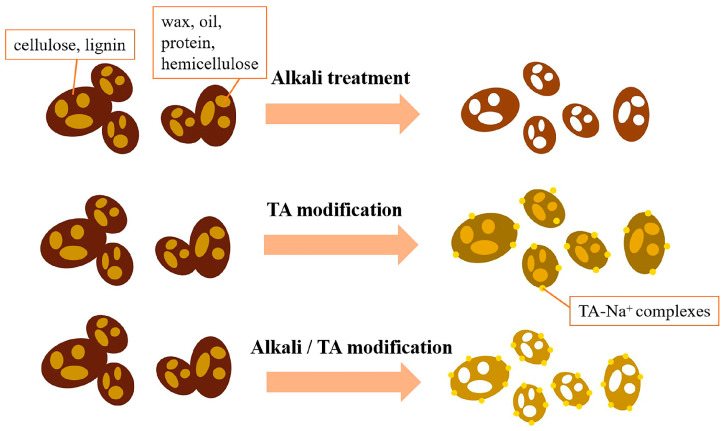
Mechanisms of alkali treatment, TA modification, and alkali/TA synergistic modification of CG.

Improvements in the tensile properties could be attributed to the improved dispersion of CGs and the enhanced physical adhesion between CG and PBAT because the crystallinity of these samples was similar [46]. The improved interfacial adhesion provides a better stress transfer from PBAT to CG, which results in a more compact fracture and better tensile properties. The mechanisms of alkali treatment, TA modification, and alkali/TA synergistic modification of CG are shown in Figure 7.

**Figure 8:**
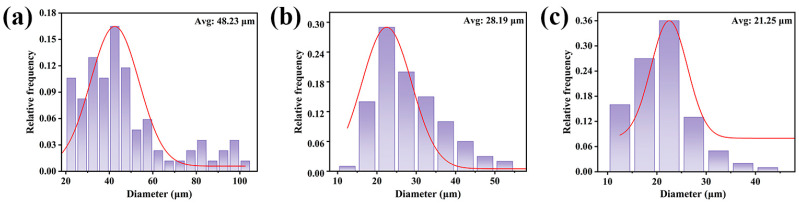
Particle size distribution of CG using different mesh sieves: (**a**) 60 mesh; (**b**) 100 mesh; and (**c**) 200 mesh.

Compared to neat PBAT, the PBAT/CG composites showed decreased tensile properties even with modified CG, which was maybe closely related to micro-sized CGs. Therefore, the effect of the CG particle size on the tensile properties of the PBAT/CG composites was also investigated. As shown in Figure 8, the CG particle size was reduced by a mechanical crusher, sieved, and finally, it acquired three types of CGs with average particle sizes of 48.23, 28.19, and 21.25 μm under different mesh sieves of 60, 100, and 200 mesh.

**Figure 9:**
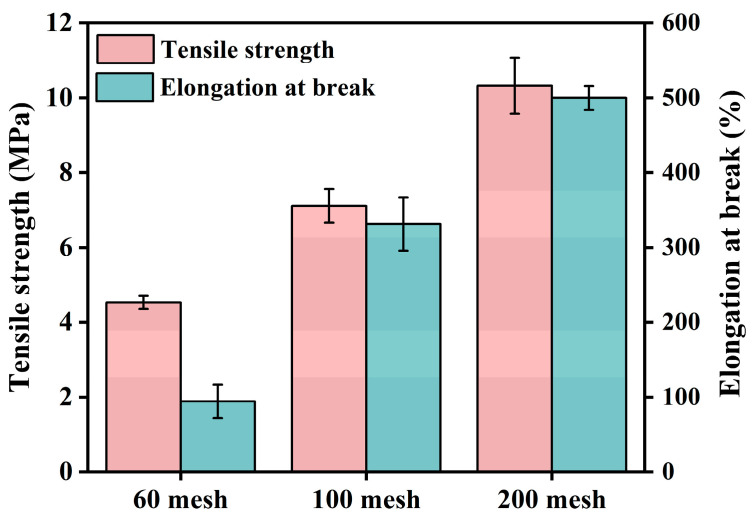
Tensile properties of PBAT/CG composites with different CG particle sizes.

The effects of particle size on the tensile properties of PBAT composites are shown in Figure 9. Fixing the CG content at 30 wt%, the tensile strength and elongation at the break of the composites with CGs with an average size of 48.23 μm (using a 60-mesh sieve) were only 4.5 MPa and 94.3%. By decreasing the particle size of CGs, the tensile strengths of composites with CGs with an average size of 28.19 μm and with CGs with an average size of 21.25 μm were 7.1 MPa and 10.3 MPa, respectively, and their elongation at break were 331.2% and 499.8%, respectively. The results confirmed that smaller CG particle sizes contribute to the tensile properties of PBAT composites. The smaller the particle size of CGs, the larger the specific surface area that facilitates effective stress transfer. In this study, CG particles processed by a 100-mesh sieve were used, considering their easier processability and yield. To further reduce the CG particle size, it would be difficult to achieve using current mechanical crusher. According to the literature, ball milling [49] and steam blasting [50] could more effectively reduce the filler size down to nano size, which may be beneficial for improving the mechanical properties of biomass composites in the future.

**Figure 10:**
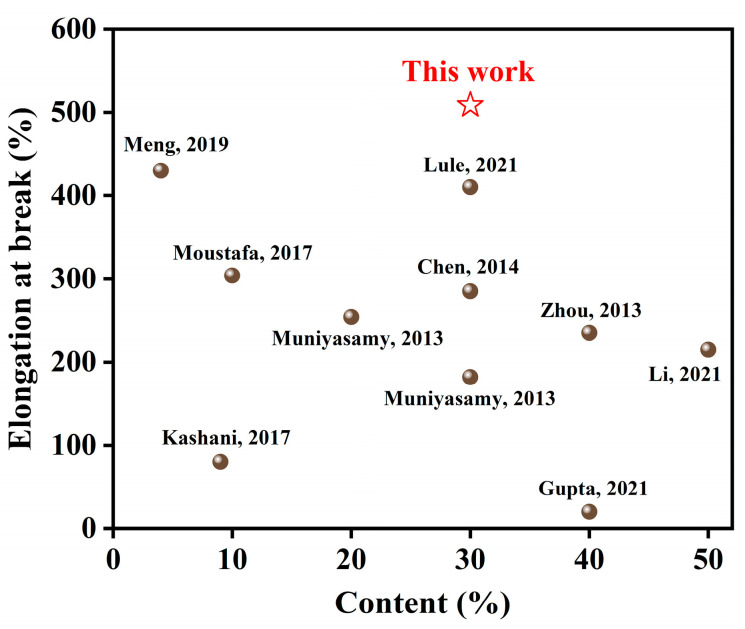
Comparison of filler content and elongation at break for PBAT-based composites [2,26,46,51,52,53,54,55,56].

Figure 10 compares the filler content and elongation at the break of the prepared PBAT/CG-OH-TA composites in this study with other PBAT-based composites [2,26,46,51,52,53,54,55,56]. In most of those studies, the loading of the incorporated biomass affected the mechanical properties, especially at high filler contents. The tensile properties of the composites could be improved by filler modification, but the used method often affects the elongation at break. However, in our study, compared to PBAT/CG composites, the obtained PBAT/CG-OH-TA composites here improved the tensile strength as well as the elongation at break. This was probably due to the increase in surface roughness of CG modified by TA, which facilitated CG distribution and the mechanical interlocking between CG and PBAT. In conclusion, the modification method used in this study preserved the excellent toughness of PBAT and provided a valuable reference for biomass-based fillers for the preparation of composites.

#### 3.2.5. Thermal Stability

**Figure 11:**
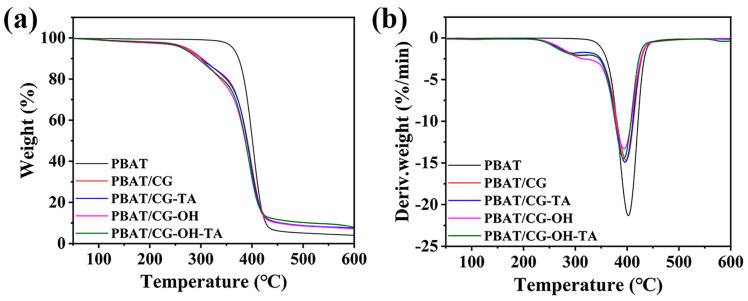
(**a**) TGA curves and (**b**) DTG curves of PBAT and PBAT/CG composites.

Figure 11 shows the thermal stability results of PBAT and corresponding PBAT/CG composites using TGA. Thermal parameters such as T_5%_, T_d-max_, and residual mass at 600 °C are concluded in Table 3. The presence of terephthalic moieties of PBAT molecular chains enables it to have a better thermal stability [2] and to decompose in a narrow temperature range. Indeed, the PBAT decomposition started at about 330 °C and was almost completed at about 490 °C. Nearly 90% of the mass loss occurred between 350 °C and 430 °C. Moreover, the maximum decomposition rate occurred at 402 °C, corresponding to a mass loss rate of 21.3%·min^−1^. All PBAT/CG composites had similar pyrolysis curves, and their thermal degradation could be divided into two steps. The first step was related to the degradation of hemicellulose (~200 °C) within CGs [22], and the second step was the same as the thermal degradation of PBAT. Therefore, the incorporation of CGs decreased the T_5%_ and T_d-max_; however, this would not affect the melt processing, since the processing temperature of PBAT is often under 200 °C.

## 4. Conclusions

In conclusion, this study introduced a novel and green method to improve the interface adhesion between CG and PBAT. Under mild conditions, TA deposited on the CG surface by forming complexes with the metal ion (Na^+^), which increased the surface’s wettability and roughness. This was confirmed using multiple techniques such as FTIR, water contact angle, SEM, and EDS. Fracture morphology and rheological property results of the prepared composites indicated that the adhesion of modified CG to PBAT was enhanced. Moreover, compared to the PBAT/CG composite, the tensile strength and elongation at the break of the PBAT/CG-OH-TA composite were enhanced by 47.0% and 53.6%, respectively. Furthermore, the addition of CGs slightly decreased the thermal stability of PBAT composites; however, this did not affect the melt processing of PBAT, which often occurred under 200 °C. This approach could provide a new method for the effective use of biomass waste as fillers, which could reduce the cost of polymer-based products by adding a large amount of biomass waste, particularly into relatively expensive biodegradable polymers.

## Figures and Tables

**Table 1:** EDS analysis of CGs (wt%).

Sample	C	O	Na	Cl	Total
CG	70.23	29.63	0.14	0	100.00
CG-TA	66.10	31.42	0.83	1.65	100.00
CG-OH	74.47	25.09	0.44	0	100.00
CG-OH-TA	58.04	31.26	1.95	8.75	100.00

**Table 2:** The specific thermal property data acquired from DSC.

Sample	*T_onset_*/°C	Δ*H_m_*/J·g^−1^	*T_c_*/°C	Δ*H_c_*/J·g^−1^	*X_c_*/%
PBAT	97.4	9.5	55.7	17.6	8.3
PBAT/CG	97.3	5.6	76.0	10.7	7.0
PBAT/CG-TA	97.2	5.8	75.6	10.5	7.3
PBAT/CG-OH	94.3	5.7	74.8	11.0	7.1
PBAT/CG-OH-TA	93.9	5.5	76.0	11.0	6.9

**Table 3:** The thermal parameters acquired from TGA and DTG.

Sample	T_5%_/°C	T_d-max_/°C		Residue/%
		T_d-max1_	T_d-max2_	
PBAT	326.9	-	402.2	4.0
PBAT/CG	272.9	305.0	398.2	7.2
PBAT/CG-TA	267.8	294.1	395.9	7.6
PBAT/CG-OH	267.0	311.8	393.7	7.2
PBAT/CG-OH-TA	264.1	303.5	393.2	8.1

## Data Availability

Not applicable.
